# Effect of *Bacillus subtilis* and *Bacillus licheniformis* supplementation in diets with low- and high-protein content on ileal crude protein and amino acid digestibility and intestinal microbiota composition of growing pigs

**DOI:** 10.1186/s40104-017-0168-2

**Published:** 2017-05-01

**Authors:** Chanwit Kaewtapee, Katharina Burbach, Georgina Tomforde, Thomas Hartinger, Amélia Camarinha-Silva, Sonja Heinritz, Jana Seifert, Markus Wiltafsky, Rainer Mosenthin, Pia Rosenfelder-Kuon

**Affiliations:** 1 University of Hohenheim, Institute of Animal Science, Emil-Wolff-Strasse 10, 70599 Stuttgart, Germany; 2 Present address: Department of Animal Science, Faculty of Agriculture, Kasetsart University, 50 Ngam Wong Wan Rd, Chatuchak, Bangkok 10900 Thailand; 3 Present address: University of Bonn, Institute of Animal Science, Endenicher Allee 15, 53115 Bonn, Germany; 4 Evonik Nutrition & Care GmbH, Rodenbacher Chaussee 4, 63457 Hanau-Wolfgang, Germany

**Keywords:** *Bacillus* spp., Growing pigs, Ileal digestibility, Microbiota, Protein levels

## Abstract

**Background:**

*Bacillus* spp. seem to be an alternative to antimicrobial growth promoters for improving animals’ health and performance. However, there is little information on the effect of *Bacillus* spp. in combination with different dietary crude protein (CP) levels on the ileal digestibility and microbiota composition. Therefore, the objective of this study was to determine the effect of *Bacillus* spp. supplementation to low- (LP) and high-protein diets (HP) on ileal CP and amino acid (AA) digestibility and intestinal microbiota composition.

**Methods:**

Eight ileally cannulated pigs with an initial body weight of 28.5 kg were randomly allocated to a row-column design with 8 pigs and 3 periods of 16 d each. The assay diets were based on wheat-barley-soybean meal with two protein levels: LP (14% CP, as-fed) and HP diet (18% CP, as-fed). The LP and HP diets were supplemented with or without *Bacillus* spp. at a level of 0.04% (as-fed). The apparent ileal digestibility (AID) and standardized ileal digestibility (SID) of CP and AA was determined. Bacterial community composition from ileal digesta was analyzed by Illumina amplicon sequencing and quantitative real-time PCR. Data were analyzed as a 2 × 2 factorial design using the GLIMMIX procedures of SAS.

**Results:**

The supplementation with *Bacillus* spp. did not affect both AID and SID of CP and AA in growing pigs. Moreover, there was no difference in AID of CP and AA between HP and LP diets, but SID of cystine, glutamic acid, glycine, and proline was lower (*P* < 0.05) in pigs fed the HP diets. The HP diets increased abundance of *Bifidobacterium* spp. and *Lactobacillus* spp., (*P* < 0.05) and by amplicon sequencing the latter was identified as predominant genus in microbiota from HP with *Bacillus* spp., whereas dietary supplementation of *Bacillus* spp. increased (*P* < 0.05) abundance of *Roseburia* spp..

**Conclusions:**

The HP diet increased abundance of *Lactobacillus* spp. and *Bifidobacterium* spp.. The supplementation of *Bacillus* spp. resulted in a higher abundance of healthy gut associated bacteria without affecting ileal CP and AA digestibility, whereas LP diet may reduce the flow of undigested protein to the large intestine of pigs.

## Background

Due to the ban of antimicrobial growth promoters in livestock feeding by the European Union in 2006 [1], probiotics are considered as an alternative for improving animals’ health and performance [2, 3]. Within this regard, *Bacillus* spp. have the ability to sporulate, thereby making them stable during thermal treatment of feed, and resistant to enzymatic digestion along the gastrointestinal tract (GIT) [4]. Thus, *Bacillus* spp. such as *Bacillus subtilis* (*B. subtilis*) and *Bacillus licheniformis* (*B. licheniformis*) are frequently supplemented to pig diets [4–6] as these two species have been listed to be added as non-toxigenic, biological supplements to livestock diets [7], and additionally, they are widely used for the large-scale industrial production of proteins including extracellular enzymes [8]. Positive effects of dietary supplementation of *B. subtilis* and *B. licheniformis* on pigs’ growth performance have been reported before [9, 10].

Activity of probiotics is influenced by diet composition [11] and variations in dietary protein supply, thereby possibly affecting microbial composition in the gut [12, 13]. Accordingly, reducing the dietary crude protein (CP) level has been reported to markedly reduce the production of potentially harmful microbial metabolites such as ammonia and amines due to the lower availability of undigested protein for microbial fermentation [14]. Thus, excessive nitrogen (N) excretion by pigs is mitigated, resulting in a decrease of environmental pollutants [15, 16]. In contrast, increasing the dietary CP intake may stimulate the proliferation of almost all bacteria groups over the entire GIT including beneficial bacteria, such as *Bifidobacterium* spp., and potentially pathogenic bacteria, such as *Bacteroides* groups [17]. Furthermore, there is increasing evidence that interactions of supplemental probiotics with dietary CP level affect the intestinal microbiome at the ileal level [18].

According to the results of previous studies [19, 20] *Bacillus* spp. enhanced the development and activities of digestive enzymes in the GIT, which was associated with a numerical increase in apparent ileal digestibility (AID) and standardized ileal digestibility (SID) of some amino acids (AA) in weaning pigs [21]. However, studies with growing pigs in which *Bacillus* spp. were supplemented to diets varying in CP content are still lacking. Therefore, the objective of the present study was to test the hypothesis, if *B. subtilis* and *B. licheniformis* supplementation to low- and high-protein diets will affect ileal CP and AA digestibility and intestinal microbiota composition in growing pigs.

## Methods

The research protocol was reviewed and approved by the German Ethical Commission for Animal Welfare, and care of the animals throughout this experiment was in accordance with guidelines issued by the Council Directive [22].

### Animals, housing, and surgical procedures

Eight pigs were obtained from the University of Hohenheim Research Station. The average initial and final body weight (BW) of the experimental animals were 28.5 ± 0.8 and 64.3 ± 1.5 kg, respectively. The pigs were housed individually in stainless steel metabolic crates (0.8 m × 1.5 m). Each metabolic crate was equipped with an infrared heating lamp and a low pressure drinking nipple which allowed free access to water. The research unit was equipped with an automated temperature control system kept at 20 °C. Until the beginning of the experiment, the pigs were fed a commercial starter diet at a daily level of 4% (as-fed)/kg of average BW (Porcigold® SMA 134, Raiffeisen Kraftfutterwerke Süd GmbH, Würzburg, Germany; 17.5% CP and 13.4 MJ metabolizable energy (ME)/kg, as-fed). After arrival at the research unit, the pigs were surgically fitted with a simple T-cannula at the distal ileum as described by Li et al. [23]. The pigs were allowed a recovery period of at least 7 d. During this period, the feed allowance was gradually increased, starting from 50 g/d the day after surgery until 1000 g/d (as-fed) were consumed.

### Experimental design, diets, and procedures

The experiment was arranged as a row-column design with 8 pigs and 3 experimental periods of 16 d each. Pigs were fed assay diets twice daily at 0700 and 1900 h at a level of 4% (as-fed)/kg of their average BW corresponding to 3 times their energy requirement for maintenance (i.e. 0.44 MJ ME/kg BW^0.75^) [24]. Pigs’ BW was determined at the beginning of each experimental period.

The assay diets were based on wheat, barley, and soybean meal with 2 protein levels resulting in a low-protein (14% CP, as-fed; LP) and a high-protein diet (18% CP, as-fed; HP). The LP diet was accomplished by blending the HP diet with 25% of native cornstarch. The contents of oil, minerals, vitamins, and titanium dioxide were the same for all diets. The *Bacillus* spp. product is comprised of a mixture of spray-dried spores of *B. licheniformis* and *B. subtilis*. The LP and HP diets were supplemented with (+) or without (-) *Bacillus* spp. at a level of 0.04% (as-fed). All assay diets were formulated (Table 1) to meet or exceed the dietary threshold levels for CP and AA according to Fan et al. [25] and NRC [26] nutrient recommendations for pigs from 25 to 50 kg BW. Vitamins and minerals were supplemented to all diets to meet or exceed NRC [26] standard, and all diets contained titanium dioxide at a level of 0.4% (as-fed basis) as an indigestible marker.

**Table 1:** Ingredient composition of assay diets, % as-fed basis

Item	High-protein	Low-protein
Barley	20.00	15.00
Wheat	51.00	38.24
Soybean meal	21.51	16.13
Oil^a^	1.50	1.50
Cornstarch^b^	2.08	25.55
Vitamins and minerals premix^c^	0.76	0.76
Sodium chloride	0.07	0.07
Monocalcium phosphate	0.66	0.66
Calcium carbonate	0.65	0.65
Vitamin E^d^	0.03	0.03
L-Lysine-HCl^e^	0.61	0.46
DL-Methionine^e^	0.22	0.16
L-Isoleucine^e^	0.03	0.02
L-Leucine^e^	0.13	0.10
L-Threonine^e^	0.22	0.17
L-Tryptophan^e^	0.01	0.01
L-Valine^e^	0.12	0.09
Titanium dioxide	0.40	0.40
*Bacillus* spp.^f^	-	-
Calculated chemical composition^g^		
Metabolizable energy, MJ/kg	13.43	14.25
Crude protein, %	18.00	14.00
Calcium, %	0.66	0.63
Available Phosphorus, %	0.27	0.25
SID^g^ Lysine, %	1.20	0.92
SID^g^ Methionine, %	0.43	0.33
SID^g^ Threonine, %	0.73	0.56

^a^Blend of rapeseed oil (75%) and soybean oil (25%)

^b^Roquette, Lestrem, France

^c^Vilomin® 18950, Deutsche VilomixTierernährung GmbH, Neuenkirchen-Vörden, Germany; provided the following quantities of minerals and vitamins per kg of diet: Ca, 1.86 g; P, 0.38 g; Na, 0.42 g; Mg, 76.00 mg; Fe, 30.40 mg (FeSO_4_·H_2_O); Cu, 3.80 mg (CuSO_4_·5H_2_O); Mn, 20.29 mg (MnO); Zn, 25.38 mg (ZnO); I, 0.51 mg (Ca(IO_3_)_2_); Se, 0.10 mg (Na_2_SeO_3_); Co, 0.06 mg (2CoCO_3_·3Co(OH)_2_·H_2_O); vitamin A, 3,040 IU; vitamin D_3_, 456 IU; vitamin E, 19.00 mg; vitamin B_1_, 0.38 mg; vitamin B_2_, 1.18 mg; vitamin B_6_, 0.95 mg; vitamin B_12_, 7.60 μg; vitamin K_3_, 0.76 mg; niacin, 4.75 mg; calcium pantothenate, 2.85 mg; folic acid, 0.19 mg; choline chloride, 57.00 mg

^d^LutavitE 50, BASF, Ludwigshafen, Germany

^e^All crystalline amino acids (AA) were supplied by Evonik Industries AG (Hanau-Wolfgang, Germany). The purity of all crystalline AA was 99%, with the exception of L-Lysine-HCl (78%)

^f^High- and low-protein diets were supplemented with or without 0.04% (as-fed) of *Bacillus* spp. product at the expense of cornstarch

^g^ *SID* standardized ileal digestibility

The assay diets were in a mash form mixed with water (1/1, w/v). During each of the 3 experimental periods, the pigs were allowed to adapt to their assay diets for 14 d before ileal digesta was collected for a total of 24 h from 0700 to 1900 h on d 15 and from 1900 on d 16 to 0700 h on d 17. Digesta collection procedure was adapted from Li et al. [23] using soft plastic bags attached to the barrel of the cannula by elastic bands. The bags were changed at least every 20 min. To minimize further bacterial fermentation 4 mL of 2.5 mol/L formic acid were added to the sampling bags and then immediately frozen at -18 °C. The individual digesta samples of each pig were pooled for each sampling period, freeze-dried, and ground to 0.5 mm prior to analyses. For analyses of intestinal microbiota composition, ileal digesta and feces samples were taken prior to the first experimental period (starter period) and on d 15 once for each experimental period. Ileal digesta and feces samples for microbial community analysis were immediately put on ice before being stored in a freezer at -80 °C for subsequent treatment and analyses.

### Chemical analyses

Official standard methods [27] were used to determine contents of proximate nutrients, neutral detergent fiber (NDF), acid detergent fiber (ADF), acid detergent lignin (ADL), and microbial numbers of *B. subtilis* and *B. licheniformis* in assay diets. The assay diets and digesta samples were analyzed for DM (method 3.1). In addition, assay diets were analyzed for ash (method 8.1); ether extract (EE; method 5.1.1 using petroleum ether), NDF assayed with a heat-stable amylase and expressed inclusive of residual ash (method 6.5.1), ADF expressed inclusive of residual ash (method 6.5.2), and ADL determined by solubilization of cellulose with sulphuric acid (method 6.5.3). Moreover, microbial numbers of *B. subtilis* and *B. licheniformis* in assay diets were determined by method 28.2.2 [27]. Nitrogen contents in assay diets and ileal digesta samples were analyzed using a gas combustion method according to official method 990.03 of the AOAC International [28] (FP-2000, Leco Corp., St Joseph, MI, US). Ethylenediaminetetraacetic acid was used as a reference standard before and after all N analyses. Crude protein contents were calculated by multiplying the content of N with 6.25. Amino acid contents in assay diets and ileal digesta samples were determined by using ion-exchange chromatography with postcolumn derivatization with ninhydrin [29]. Tryptophan was determined by HPLC with fluorescence detection (extinction 280 nm, emission 356 nm), after alkaline hydrolysis with barium hydroxide octahydrate for 20 h at 110 °C according to the procedure as outlined by Commission Directive [30]. The titanium dioxide content in the assay diets and ileal digesta samples was performed according to the procedure described by Brandt and Allam [31].

### DNA extraction of ileal digesta and feces samples

Genomic DNA was extracted from 250 mg ileal digesta and feces using Fast DNA Spin Kit for Soil (MP Biomedicals GmbH, Heidelberg, Germany). Extraction procedure was performed with slight modifications to manufacturer’s instructions as described by Burbach et al. [32].

### Amplicon sequencing analysis

Illumina amplicon sequencing libraries of the V1-2 region of the 16S rRNA gene was performed similar to procedures described previously [33]. Library preparation, however, was modified as follows: the V1-2 region was amplified with a 27 F-modified forward primer (AGRGTTHGATYMTGGCTCAG) in a 20 μL reaction. 1 μL of this first PCR was used as template in a second PCR using multiplexing and indexing primers as described previously [33]. Amplicons were verified by agarose gel electrophoresis and normalized using SequalPrep^TM^ Normalization Plate Kit (Invitrogen, Thermo Fisher Scientific, Waltham, USA). Libraries were pooled by index, purified with MinElute PCR Purification Kit (Qiagen, Hilden, Germany), quantified with Qubit^®^ 2.0 Fluorometer (Invitrogen) and sequenced on Illumina MiSeq platform using 250 bp paired end sequencing chemistry. All analyzed samples comprised around 2.8 million reads, with an average of 43,646 reads per sample. Reads were quality filtered, assembled and aligned using Mothur pipeline [34]. UCHIME was used to find possible chimeras and reads were clustered at 97% identity into 2601 operative taxonomic units (OTU). The closest representative was manually identified with seqmatch from RDP [35]. Sequences classified as Chloroplast/Cyanobacteria were removed from OTU dataset as it was assumed that they represent undigested plant material. Sequences were submitted to European Nucleotide Archive under the accession number PRJEB14413 (http://www.ebi.ac.uk/ena/data/view/PRJEB14413).

### Quantitative real time PCR

Quantitative real-time PCR (qPCR) was used to analyze the following bacteria groups in the ileal digesta samples: Total bacteria, *Lactobacillus* spp*.*, *Bifidobacterium* spp., *Roseburia* spp., *Enterobacteriaceae*, *Bacteroides-Prevotella-Porphyromonas* group, *Clostridium* Cluster IV, and *Bacillus* spp.. All used primers were selected from literature and are listed in Table 2. Optimization of primer conditions was done in order to determine optimal annealing temperatures and primer concentrations by running a standard PCR with diverse primer concentrations (200 nmol/L, 400 nmol/L, 600 nmol/L) and a temperature gradient from 55.0 °C to 65.0 °C. According to melt curves on standard PCR and the agarose gel electrophoresis results, optimal primer concentration and annealing temperature was set for each primer.

**Table 2:** Oligonucleotide primers used for real-time PCR

Target group	Item	Oligonucleotide sequence (5′→3′)	Primer conc., nmol/L	Annealing temp., °C	Product size, bp	Reference
Total bacteria	Forward	GTGSTGCAYGGYYGTCGTCA	600	52	147	Fuller et al. [80]
	Reverse	ACGTCRTCCMCNCCTTCCTC				
*Lactobacillus* spp*.*	Forward	AGAGGTAGTAACTGGCCTTTA	400	59	391	Malinen et al. [81]
	Reverse	GCGGAAACCTCCCAACA				
*Bifidobacterium* spp.	Forward	TCGCGTCYGGTGTGAAAG	400	59	243	Rinttilä et al. [82]
	Reverse	CCACATCCAGCRTCCAC				
*Roseburia* spp.	Forward	AGGCGGTACGGCAAGTCT	400	59	353	Veiga et al. [83]
	Reverse	AGTTTYATTCTTGCGAACG				Rinttilä et al. [82]
*Enterobacteriaceae*	Forward	CATTGACGTTACCCGCAGAAGAAGC	200	59	195	Bartosch et al. [84]
	Reverse	CTCTACGAGACTCAAGCTTGC				
*Clostridium* Cluster IV	Rflbr730F	GGCGGCYTRCTGGGCTTT	400	65	147	Ramirez-Farias et al. [85] Lay et al. [86]
	Clep866mR§	CCAGGTGGATWACTTATTGTGTTAA				
*Bacteroides-Prevotella-Porphyromonas*	Forward	GGTGTCGGCTTAAGTGCCAT	600	58	140	Rinttilä et al. [82]
	Reverse	CGGAYGTAAGGGCCGTGC				
*Bacillus* spp.	Forward	CCTACGGGAGGCAGCAGTAG	600	59	78	Fernández-No et al. [87]
	Reverse	GCGTTGCTCCGTCAGACTTT				

Standard curves for each primer were designed using serial dilutions of the purified and quantified PCR products generated by standard PCR and genomic DNA from pig feces. The PCR products were checked by agarose gel electrophoresis (2% agarose) to ensure correct primer specific products. Quantity of purified PCR amplification products was determined using Qubit^®^ 2.0 Fluorometer (Invitrogen).

Quantification was carried out using the CFX Connect™ Real-Time System (Bio-Rad Laboratories GmbH, Munich, Germany), associated with the Bio Rad CFX Manager™ Software 3.1 (Bio-Rad Laboratories GmbH, Munich, Germany). All samples were determined in duplicate and all standards were pipetted in triplicate on each plate. The order of samples and standards on the plates was randomized. The reaction mixture for each bacterial group consisted of 10 μL of KAPA SYBR FAST (PEQLAB Biotechnologie GmbH, Erlangen, Germany), 1 μL template DNA (ileal digesta samples and standards), the optimized primer concentrations of forward and reverse primers (Table 2), and was filled up to a total volume of 20 μL with PCR grade water (Carl Roth GmbH, Karlsruhe, Germany). Amplification conditions were: activation of polymerase at 95.0 °C for 3 min, followed by 40 cycles consisting of denaturation at 95.0 °C for 5 s, primer annealing for 20 s (at optimized temperatures, Table 2), and extension at 72.0 °C for 1 s. Subsequently, a final elongation step at 72.0 °C for 1 min followed. The melt curve was obtained by stepwise (0.5 °C) increase of temperature from 55 °C to 95 °C. Results were reported as log_10_ 16S rRNA gene copies/g digesta.

### Calculations

The AID of CP and AA in the assay diets was calculated according to the following equation:

$$ {\mathrm{A}\mathrm{ID}}_{\mathrm{D}} = \left[1\ \hbox{--}\ \left({\mathrm{I}}_{\mathrm{D}} \times {\mathrm{A}}_{\mathrm{I}}\right)\ /\ \left({\mathrm{A}}_{\mathrm{D}} \times {\mathrm{I}}_{\mathrm{I}}\right)\right] \times 100\ \% $$

where AID_D_ = AID of CP or AA in the assay diet (%), I_D_ = marker content in the assay diet (g/kg DM), A_I_ = CP or AA content in ileal digesta (g/kg DM), A_D_ = CP or AA content in the assay diet (g/kg DM), and I_I_ = marker content in ileal digesta (g/kg DM).

According to Stein et al. [36] and Jansman et al. [37], the basal ileal endogenous loss of CP and AA (IAA_end_) is considered to be constant among groups of pigs, and therefore, mean values for IAA_end_ [37] can be used for transformation of AID into their SID values.

The SID of CP and AA in assay diets was estimated according to the following equation:

$$ {\mathrm{SID}}_{\mathrm{D}} = {\mathrm{A}\mathrm{ID}}_{\mathrm{D}} + \left({\mathrm{IAA}}_{\mathrm{end}}/\ {\mathrm{A}}_{\mathrm{D}}\right) \times 100\ \% $$

where SID_D_ = SID of CP or AA in the assay diet (%).

### Statistical analyses

Homogeneity of variances and normal distribution of the data were confirmed using the UNIVARIATE procedure of SAS (SAS Inst., Inc., Cary, NC). Data were analyzed as a 2 × 2 factorial using the GLIMMIX procedures of SAS. The model included the protein level, probiotic supplementation, and the interactive effects of protein level and probiotic supplementation as the fixed effects, and pig and period as the random effects. In case of interaction, the significant differences between treatments based on a *t*-test were set at α = 0.05 using the algorithm for letter-based representation of all pair-wise comparisons according to Piepho [38]. For microbiota analyses, bacterial 16S rRNA gene copy numbers in pre-treatment period was considered as covariate. Least squares means and standard error of the means are presented, and a probability level of *P* < 0.05 was considered to be statistically significant, whereas a *P* < 0.10 was considered to constitute a tendency.

Illumina amplicon sequencing data were analyzed using statistic software PRIMER (v.6.1.16, PRIMER-E; Plymouth Marine Laboratory, Plymouth, UK) [39]. Samples were standardized by total and resemblance matrix was calculated using Bray-Curtis coefficient. Overall community structures were explored by nonmetric multidimensional scaling (MDS). One way analysis of similarity (ANOSIM) and permutational multivariate analysis of variance (PERMANOVA) were used to evaluate similarity between different dietary groups, different protein levels and probiotic treatments, and a probability level of *P ≤* 0.05 was considered to be significant different. The ANOSIM *R* values range from -1 to 1; the farer from zero the more distinct and the closer to zero the more similar are the compared groups. Variables contributing to observed differences were identified by similarity percentages routine. The bacterial families contributing to overall 70% of dissimilarities among treatment groups were considered to be the most important and their abundance data were graphically plotted according to a color key from zero to maximal abundance. Shannon index was used to measure diversity in bacterial communities from different sample groups, taking into account the number of OTUs and the proportion of each OTU. A Mantel-type test (RELATE) on Bray-Curtis matrices was used to quantify the correlation between results from bacterial community analysis. To enable comparison between amplicon sequencing and qPCR approaches, RELATE routine was run on untransformed datasets, restricted to bacteria groups targeted by qPCR primers and the generated Spearman Rho was considered to be significant if *P* ≤ 0.05.

## Results

All pigs remained healthy throughout the experiment and readily consumed their daily feed allowances. The analyzed CP and AA contents of the assay diets and microbial numbers of *B. subtilis* and *B. licheniformis* in assay diets are presented in Table 3. As expected, CP and AA contents in LP were approximately 76.5 and 76.6% that of HP, respectively. The contents of ash, EE, NDF, ADF, and ADL in the HP diets were also greater than in the LP diets. The *Bacillus* spores determined in the experimental diets amounted to 1.54 × 10^9^ CFU/kg feed for HP + and LP + diets, whereas HP - and LP - diets contained 0.02 × 10^9^ and 0.04 × 10^9^ CFU/kg feed, respectively.

**Table 3:** Analyzed chemical composition and *Bacillus* cell numbers in assay diets

	High-protein		Low-protein	
Item	-	+	-	+
Dry matter, %	88.6	88.7	88.3	88.6
Crude protein, % DM	20.6	20.3	15.2	16.1
Ash, % DM	6.1	6.0	5.2	5.3
Ether extract, % DM	3.7	3.6	3.2	3.3
Neutral detergent fiber, % DM	12.7	13.1	10.1	10.5
Acid detergent fiber, % DM	7.0	6.6	5.4	5.1
Acid detergent lignin, % DM	1.1	0.9	0.8	0.8
Indispensable amino acids, % DM				
Arginine	1.26	1.25	0.93	0.99
Histidine	0.46	0.46	0.35	0.36
Isoleucine	0.80	0.80	0.61	0.63
Leucine	1.53	1.53	1.15	1.19
Lysine	1.48	1.50	1.12	1.12
Methionine	0.50	0.51	0.38	0.37
Phenylalanine	0.95	0.95	0.69	0.74
Threonine	0.91	0.91	0.68	0.70
Tryptophan	0.27	0.27	0.20	0.21
Valine	1.02	1.01	0.76	0.79
Dispensable amino acids, % DM				
Alanine	0.80	0.79	0.60	0.63
Aspartic acid	1.73	1.71	1.29	1.37
Cystine	0.33	0.33	0.25	0.26
Glutamic acid	4.08	4.03	3.05	3.20
Glycine	0.81	0.80	0.61	0.64
Proline	1.32	1.31	0.99	1.04
Serine	0.93	0.91	0.69	0.73
*Bacillus* cell numbers, CFU/kg feed				
*Bacillus subtilis*	0.022 × 10^9^	0.860 × 10^9^	0.038 × 10^9^	0.970 × 10^9^
*Bacillus licheniformis*	<0.002 × 10^9^	0.680 × 10^9^	0.006 × 10^9^	0.570 × 10^9^

The AID and SID of CP and AA in the assay diets are shown in Tables 4 and 5, respectively. The supplementation with *Bacillus* spp. did not affect both AID and SID of CP and AA. Furthermore, there was no difference in AID of CP and AA between HP and LP diets, but SID of cystine, glutamic acid, glycine, and proline was lower (*P* < 0.05) in the HP diets than in the LP diets. Moreover, SID of CP, alanine, aspartic acid, and serine also tended to be lower (*P* < 0.10) in the HP diets. However, no interactions between CP level and *Bacillus* spp. supplementation could be observed for AID and SID of CP and AA in the present study.

**Table 4:** Apparent ileal digestibility of crude protein and amino acids of the assay diets^a^

	High-protein		Low-protein		SEM	*P*-value		
Item	-	+	-	+		P^1^	B^2^	P × B^3^
Crude protein	76.4	75.4	80.0	76.6	2.09	0.273	0.310	0.573
Indispensable amino acids								
Arginine	85.4	84.8	87.1	84.7	1.35	0.563	0.286	0.534
Histidine	80.4	79.2	82.9	79.7	1.70	0.398	0.211	0.551
Isoleucine	79.3	78.6	82.5	79.1	1.96	0.356	0.313	0.513
Leucine	81.1	80.3	83.8	80.6	1.73	0.389	0.269	0.501
Lysine	84.8	84.3	87.5	84.2	1.38	0.351	0.194	0.327
Methionine	89.7	89.3	91.4	89.0	1.03	0.492	0.200	0.349
Phenylalanine	78.6	78.1	82.0	79.0	1.99	0.295	0.386	0.522
Threonine	76.6	75.7	79.5	75.3	2.09	0.546	0.235	0.446
Tryptophan	75.1	73.2	78.1	73.8	2.47	0.470	0.226	0.628
Valine	78.8	78.9	81.9	78.1	1.93	0.403	0.232	0.471
Dispensable amino acids								
Alanine	70.6	68.9	75.5	71.0	2.66	0.197	0.257	0.605
Aspartic acid	74.6	73.4	78.6	74.7	2.32	0.263	0.284	0.566
Cystine	74.3	72.6	78.9	74.5	2.33	0.176	0.200	0.568
Glutamic acid	86.4	85.7	88.9	86.9	1.22	0.148	0.267	0.601
Glycine	65.5	63.4	70.1	64.3	2.91	0.352	0.188	0.521
Proline	82.7	81.4	85.3	82.2	1.64	0.312	0.197	0.611
Serine	76.7	75.2	79.7	76.3	2.10	0.341	0.244	0.665

^1^ *P*-value of protein level

^2^ *P*-value of probiotic supplementation with *Bacillus* spp.

^3^ *P*-value of interaction between protein level and probiotic supplementation with *Bacillus* spp.

^a^LS means and standard error of the means, %

**Table 5:** Standardized ileal digestibility of crude protein and amino acids of the assay diets^a^

	High-protein		Low-protein		SEM	*P*-value		
Item	-	+	-	+		P^1^	B^2^	P × B^3^
Crude protein	82.1	81.3	87.8	83.9	2.09	0.063	0.274	0.488
Indispensable amino acids								
Arginine	88.5	87.9	91.3	88.7	1.35	0.210	0.254	0.474
Histidine	84.5	83.4	88.4	84.9	1.70	0.128	0.187	0.492
Isoleucine	84.0	83.3	88.8	85.2	1.96	0.110	0.289	0.474
Leucine	84.3	83.5	88.0	84.7	1.73	0.164	0.255	0.472
Lysine	87.5	87.0	91.1	87.8	1.38	0.126	0.192	0.333
Methionine	91.9	91.5	94.3	92.0	1.03	0.166	0.201	0.363
Phenylalanine	82.2	81.7	86.9	83.6	1.99	0.112	0.348	0.474
Threonine	83.3	82.4	88.5	84.1	2.09	0.114	0.212	0.410
Tryptophan	80.3	78.4	85.0	80.3	2.47	0.195	0.202	0.584
Valine	84.2	83.2	89.0	85.0	1.93	0.106	0.218	0.436
Dispensable amino acids								
Alanine	76.9	75.2	83.9	79.0	2.66	0.056	0.232	0.549
Aspartic acid	79.3	78.2	85.0	80.7	2.32	0.094	0.259	0.509
Cystine	80.7	78.9	87.5	82.5	2.33	0.037	0.166	0.499
Glutamic acid	89.5	88.8	93.1	90.8	1.22	0.034	0.242	0.539
Glycine	76.8	74.8	85.3	78.7	2.91	0.045	0.159	0.436
Proline	91.4	90.1	96.8	93.2	1.64	0.018	0.158	0.486
Serine	84.1	82.6	89.6	85.6	2.10	0.055	0.209	0.547

^1^ *P*-value of protein level

^2^ *P*-value of probiotic supplementation with *Bacillus* spp.

^3^ *P*-value of interaction between protein level and probiotic supplementation with *Bacillus* spp.

^a^LS means and standard error of the means, %

**Fig. 1:**
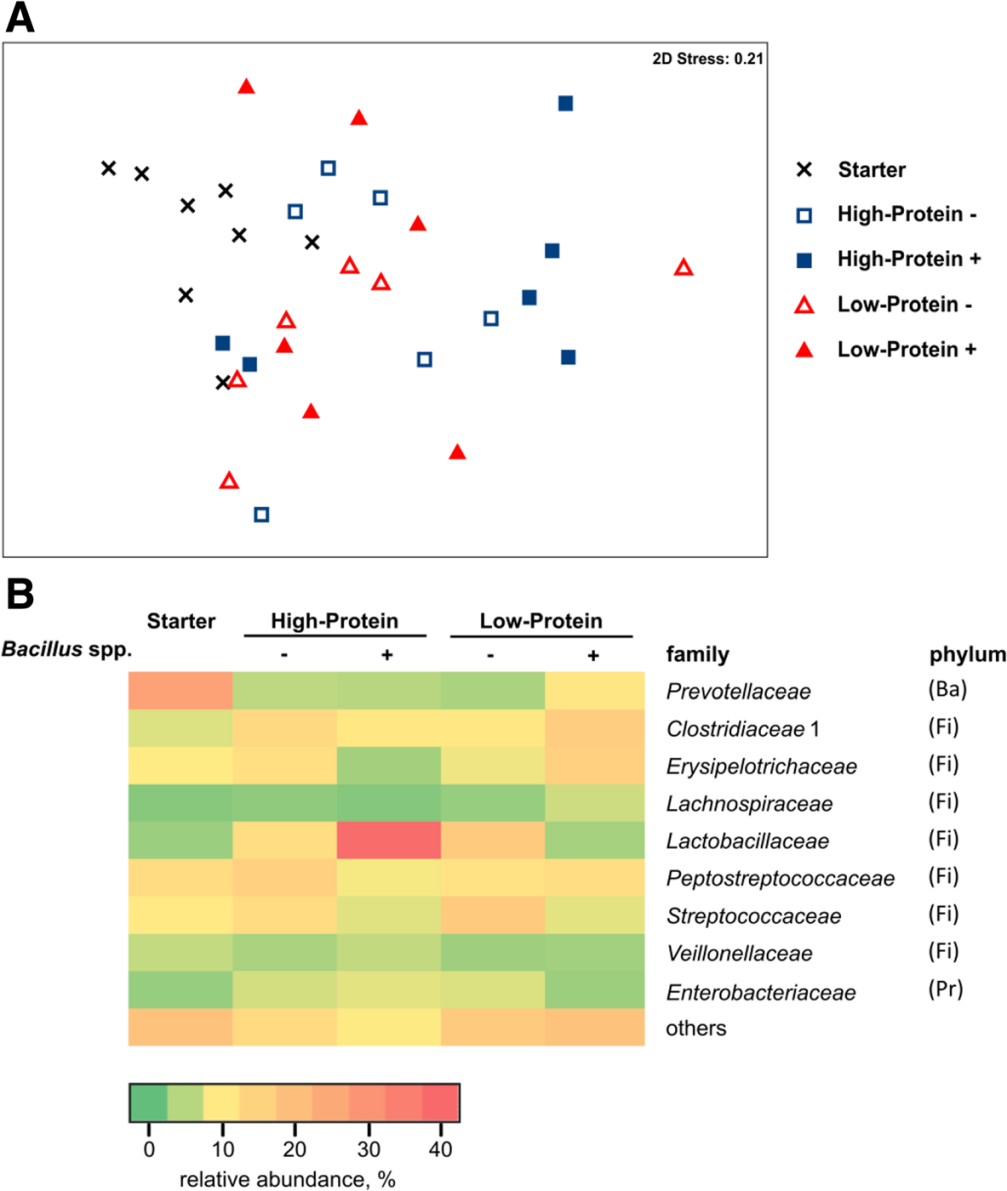
Microbiota composition in ileal digesta samples from pigs fed starter diet and assay diets. **a** Multidimensional scaling plot based on Bray Curtis similarity matrix of 16S rDNA sequence data from ileal digesta. **b** Abundance plot of most important bacterial families in overall microbiota structure of ileal digesta. Phyla: *Firmicutes* (Fi), *Bacteroidetes* (Ba), *Proteobacteria* (Pr)

The overall structure in bacterial communities from ileal digesta was evaluated by 16S rRNA gene amplicon sequencing. Analysis of similarity revealed significant differences in microbiota composition due to different dietary treatments (*P* = 0.05), but a statistic *R* value close to zero (*R* = 0.176) suggests a weak separation of the different treatment groups (Fig. 1a).

When compared with the starter period, bacterial communities were different (*P* < 0.01) between dietary treatments. Within assay diets, however, there were no effects (Table 6).

**Table 6:** Results from PERMANOVA test for dietary effect on 16S rRNA sequencing data from ileal digesta

Source	Degrees of freedom	Sum of squares	Mean square	Pseudo-F	*P*(perm)	Unique perms
P^1^	1	2022.4	2022.4	0.770	0.638	998
B^2^	1	1340.1	1340.1	0.511	0.901	998
P × B^3^	1	2691.9	2691.9	10.255	0.424	999
Res	20	52,497	2624.8			
Total	23	58,551				

^1^ *P*(perm)-value of protein level

^2^ *P*(perm)-value of probiotic supplementation with *Bacillus* spp.

^3^ *P*(perm)-value of interaction between protein level and probiotic supplementation with *Bacillus* spp.

Taxonomical composition of ileal digesta samples demonstrated some variation among dietary treatments. At phylum level, the bacterial communities were dominated by *Firmicutes* and *Bacteroidetes*. Within the assay diets from periods 1 to 3, the relative abundance of *Firmicutes* was higher than *Bacteroidetes* when compared to the starter period. The reduction of *Bacteroidetes* was mainly due to lower abundance of *Prevotellaceae*, with an average abundance of 27% in the starter diet compared to 5% in the HP diets, 4% in LP - and 11% in LP +. Nine bacterial families contributed to the overall dissimilarities among microbiota structure in ileal digesta samples of different dietary treatments (Fig. 1b). Ileal microbiota from dietary treatments without probiotic supplementation consisted mainly of *Peptostreptococcaceae*, *Clostridiaceae* 1, *Streptococcaceae*, *Lactobacillaceae* and *Erysipelotrichaceae* with even proportions, except for *Peptostreptococcaceae* and *Streptococcaceae* being the predominant family in the HP and LP treatment, respectively. *Streptococcus alactolyticus* accounted for 15% of total microbiota in samples of LP - treatment. Compared to this, ileal digesta samples from LP + were enhanced in *Clostridiaceae* 1, *Erysipelotrichaceae* and *Prevotellaceae*. In HP +, the bacterial composition was dominated by *Lactobacillaceae*, with an average abundance of 40%. Here, an uncultured *Lactobacillus* from porcine intestine (relative abundance of 21.5%) and *Lactobacillus amylovorus* (14.2%) were the predominant species.

*Lactobacillus* spp. and other bacteria groups of interest were quantified in ileal digesta by qPCR (Table 7). Mantel test showed a significant correlation between the two approaches, sequencing and qPCR (Rho = 0.852, *P* < 0.01), thus confirming that both methodological approaches resulted in comparable results. The HP diets increased abundance of *Lactobacillus* spp*.* and *Bifidobacterium* spp. (*P* < 0.05). No effects of CP content on ileal gene copy numbers of total bacteria, *Roseburia* spp., *Enterobacteriaceae*, *Bacteroides*-*Prevotella*-*Porphyromonas*, *Clostridium* cluster IV and *Bacillus* spp. were found. Likewise, no significant effect of supplementation of *Bacillus* spp. was observed for ileal gene copy numbers of total bacteria, *Lactobacillus* spp., *Bifidobacterium* spp., *Enterobacteriaceae*, *Clostridium* cluster IV and *Bacillus* spp.. However, dietary supplementation of *Bacillus* spp. increased (*P* < 0.05) abundance of *Roseburia* spp., while it tended (*P* < 0.10) to promote *Bacillus* spp. and total bacteria. Furthermore, there was an interaction (*P* < 0.05) of protein level and *Bacillus* spp. supplementation for ileal gene copy numbers of *Bacteroides*-*Prevotella*-*Porphyromonas*. The LP + resulted in higher (*P* < 0.05) abundance of *Bacteroides*-*Prevotella*-*Porphyromonas* than the LP -, but did not differ from the HP diets.

**Table 7:** Ileal gene copy numbers^a^ in ileal digesta of growing pigs

	High-protein		Low-protein		SEM	*P*-value		
Item	-	+	-	+		P^1^	B^2^	P × B^3^
Total bacteria	8.9	9.1	8.4	9.1	0.30	0.286	0.070	0.226
*Lactobacillus* spp.	7.9	8.8	6.9	7.1	0.44	0.002	0.109	0.279
*Bifidobacterium* spp.	6.2	6.4	5.3	6.0	0.32	0.024	0.179	0.354
*Roseburia* spp.	7.1	7.3	6.5	7.7	0.33	0.834	0.033	0.111
*Enterobacteriaceae*	7.7	7.9	7.4	8.3	0.43	0.836	0.139	0.274
*Bacteroides*-*Prevotella*-*Porphyromonas*	8.1^b,c^	8.2^b,c^	7.6^c^	8.6^b^	0.26	0.968	0.013	0.042
*Clostridium* cluster IV	5.6	5.7	5.2	5.9	0.30	0.735	0.185	0.324
*Bacillus* spp.	8.0	8.3	7.5	8.1	0.23	0.100	0.054	0.498

^1^ *P*-value of protein level

^2^ *P*-value of probiotic supplementation with *Bacillus* spp.

^3^ *P*-value of interaction between protein level and probiotic supplementation with *Bacillus* spp.

^a^log_10_ 16S rRNA gene copies/g digesta (LS means and standard error of the means)

^b,c^Within a row, LS means with a common superscript are not different at α = 0.05

**Fig. 2:**
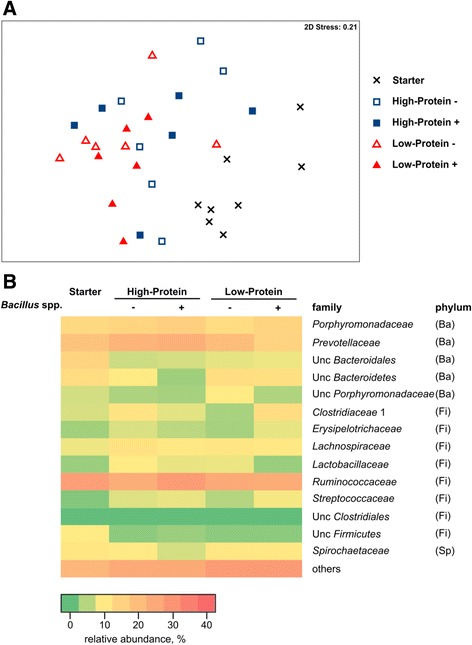
Microbiota composition in fecal samples from pigs fed starter diet and assay diets. **a** Multidimensional scaling plot based on Bray Curtis similarity matrix of 16S rDNA sequence data from fecal samples. **b** Abundance plot of most important bacterial families in overall microbiota structure of feces. Phyla: *Firmicutes* (Fi), *Bacteroidetes* (Ba), *Spirochaetes* (Sp)

The analysis of fecal microbiota by 16S rRNA gene amplicon sequencing showed no statistical effect on overall community structure. However, feces microbiota from each assay diet revealed to be significant different to that from the starter period (*P* < 0.01; Fig. 2a). At family level, *Prevotellaceae* exhibited the strongest impact on these dissimilarities (Fig. 2b), with *Prevotella* being the predominant genus. The average abundances of *Prevotella* showed slight variations for treatment groups with different protein levels; starter (15%), LP diets (13%), and HP diets (19%).

**Fig. 3:**
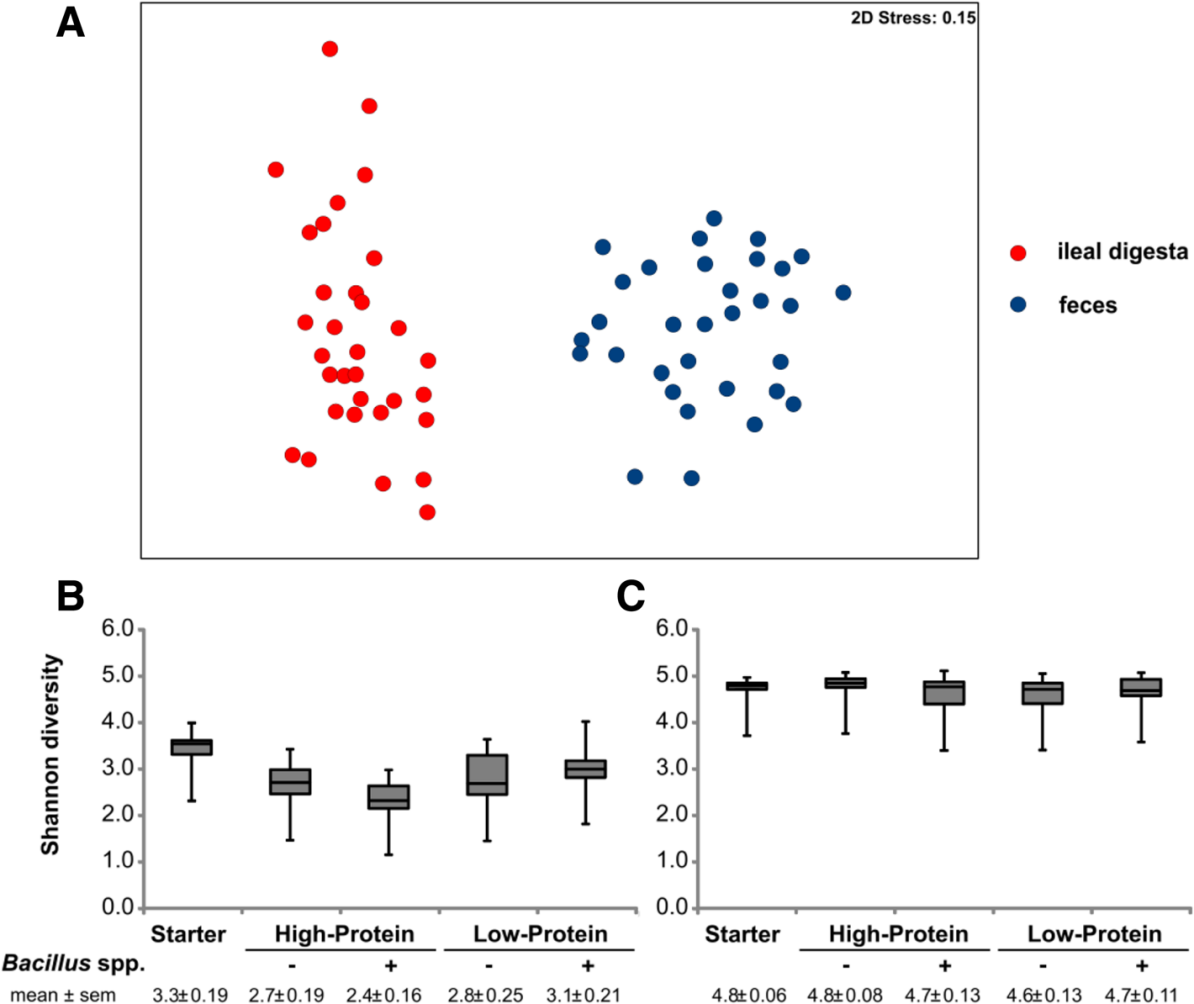
Comparison of microbiota from ileal digesta and feces. **a** MDS plot based on Bray-Curtis similarity matrix of all samples from ileal digesta and feces. **b** Shannon diversity calculated on operative taxonomic units data from ileal digesta samples (**c**) and from fecal samples

Comparing sequencing results from porcine ileal digesta and feces revealed distinct differences in bacterial communities structure (*R* = 0.924, *P* < 0.01) (Fig. 3a). Samples from ileal digesta showed a lower diversity compared to feces (Shannon index in average 2.9 vs. 4.7) (Fig. 3b and c). Mainly *Streptococcus alactolyticus* contributed to the dissimilarity with an average abundance of 9.7% in ileal digesta compared to 2.0% in feces. At family level differences were mainly due to *Lactobacillaceae* and *Ruminococcaceae*. The abundance of *Lactobacillaceae* was higher in ileal digesta (16%) than in feces (2%), and contrary the abundance of *Ruminococcaceae* was higher in feces (23%) than in ileal digesta (0.5%).

## Discussion

According to previous studies, *B. subtilis* and *B. licheniformis* produce extracellular enzymes including proteases and α-amylase [19, 20], which may enhance nutrient digestibility resulting in improved feed conversion in finisher pigs [40]. In addition, *B. subtilis* exceeds *B. licheniformis* in production of glycosyl hydrolases [4], which assist in the hydrolysis of glycosidic bonds in complex sugars. However concerning antibiotic resistance, which is considered to be an important key requirement for probiotics, a higher concentration of antibiotics is tolerated by *B. licheniformis* than by *B. subtilis* [4]. Recently, probiotic characteristics were described for spores of *B. subtilis*, although interactions with porcine epithelial cells are not understood so far [4]. For example, the supplementation of *B. subtilis* to a soybean meal diet showed slight improvements in AID and SID of some AA in weaning pigs as described by Kim et al. [21]. However, in the present study, there was no improvement in AID and SID of CP and AA in growing pigs fed diets supplemented with *B. subtilis* and *B. licheniformis*. Similarly, previous studies [6, 41] failed to demonstrate that the inclusion of *B. subtilis* and *B. licheniformis* in diets would affect apparent total tract digestibility of CP in growing-finishing pigs. The lack of probiotic treatment effects may be due to low quantity of the supplemented bacterial species in porcine intestine, as in treatments with probiotic supplementation the *Bacillus* spp. numbers were not significantly higher compared to numbers in treatments without probiotic supplementation. The gene copy numbers of *Bacillus* spp. in the treatments without probiotic supplementation correspond to results of a study by Dowd et al. [42] on *Bacillus* spp. in the ileum of piglets using 16S rRNA gene sequencing. In addition to the qPCR results, further *Bacillus* species (*B. pumilus* and *B. cereus*) were identified by amplicon sequencing. Operative taxonomic units corresponding to *Bacillus* genus appeared in very low abundance (<1%), and were present in samples with and without *Bacillus* spp. supplementation. These results are in accordance with previous studies demonstrating the ability of germinated *Bacillus* spores to proliferate in mammal GIT, even if only at a low rate [5], and therefore might not be persistent [43].

Positive effects of diets supplemented with *B. subtilis* and *B. licheniformis* on feed conversion in pigs have been reported before [40, 44], however, the underlying mechanisms of *Bacillus*’ probiotic activity are little understood, and may be attributed to competitive adhesion and immunomodulation by *Bacillus* spores or to enzymes and other substances produced by the germinated, vegetative cells of *Bacillus* [5]. Notably, probiotic supplements may be more effective under stress such as practical field conditions [45, 46]. This might be one reason for the missing effect of *Bacillus* spp. supplementation on digestibility values in the present study, as pigs were individually housed and kept in a clean environment under optimal temperature and minimal stress conditions. Furthermore, the age of pigs may be associated with probiotic efficacy [47]. The use of probiotics tended to be more effective in early age of pigs rather than the growing period [48, 49]. In the present study, grower pigs (13- and 20-week old at the initial and final BW, respectively) fed diets supplemented with *Bacillus* spp. did not show any differences in ileal digestibility of CP and AA. It has been suggested that increasing age may be a contributing factor in building up the complexity of the microbial community [50] with growing pigs being more resistant to intestinal disorders than young pigs [51].

Dietary content of CP has been reported to be associated with AID due to the variation in endogenous CP and AA losses in ileal digesta [52]. Previous research [53] suggests that AID shows segmented quadratic with plateau relationships as the level of CP and AA in the diet increased from 4 to 24% (as-fed). Alternatively, SID has been widely accepted to overcome this problem by correcting AID values for basal endogenous losses of CP and AA [54]. In general, SID values are higher in comparison to their corresponding AID values as the basal endogenous losses of CP and AA are subtracted from ileal CP and AA outflow [36]. In the present study, SID of some AA was lower in HP diets than in LP diets. Apparently, higher fiber contents in HP diets, associated with enhanced secretion of endogenous AA [55, 56], may have contributed to higher rate of digesta passage in the digestive tract of pigs [57], thereby, decreasing SID values. This is confirmed by the results of a recent study [58], where SID of CP and most AA decreased linearly with increasing dietary CP from 6.8 to 21.4% (as-fed) due to the greater NDF and ADF contents.

The higher numbers of *Lactobacillus* spp. and *Bifidobacterium* spp. in ileal digesta of HP treatments are in agreement with a recent study by Rist et al. [17], where piglets fed high dietary CP levels showed an increased growth and proliferation of lactic-acid bacteria in ileal digesta. As content of soybean meal in the present study was greater in HP than in LP diets, enhanced availability of fermentable carbohydrates in the small intestine can be suggested, thereby stimulating ileal growth of lactobacilli and bifidobacteria [17]. Furthermore, HP diets could increase the availability of free AA in the small intestine [17], contributing much more preformed AA of dietary and endogenous origin to bacterial growth in the upper part of the digestive tract than microbial *de novo* synthesis of AA [13]. Furthermore, analysis of overall microbiota composition in ileal digesta by amplicon sequencing supported an increasing effect on *Lactobacillus* proportion upon feeding of HP + diets. The presence of *Lactobacillus* spp. and *Bifidobacterium* spp. in the GIT has been reported to be beneficial for the host animal [17] due to their ability for bacteriocin production [59]. Moreover, proliferation of pathogenic bacteria may be inhibited through the production of short-chain fatty acids (SCFA) and lactic acid, being associated with a lower pH, causing a hostile environment for some acid-sensitive bacteria strains [60]. The presented sequencing results for *Lactobacillus* spp. are supported by qPCR results, which revealed a higher number of *Lactobacillus* gene copies in HP diets. The identified *Lactobacillus* spp. were dominated by an uncultured bacterium, previously isolated from porcine intestine [61], and the species *L. amylovorus. L. amylovorus* is a synonym expression for *Lactobacillus sobrius*, which is characterized by amylolytic activity, and being previously identified with high prevalence in porcine intestine [62–65]. Application of an oral probiotic mixture including a *L. amylovorus* strain has been shown to promote growth performance of pigs [66]. In general, the enhancement of potential beneficial *Lactobacillus* spp. is considered to promote gut health. However, the above described supporting effect of HP + diet on abundance of *Lactobacillus* caused a reduced community diversity compared to microbiota from ileal digesta of other dietary treatments. A high diversity in intestinal microbiota might be preferable to cope effectively with potential challenging conditions [67].

Regardless of dietary protein level, the supplementation of *B. subtilis* and *B. licheniformis* had a stimulating effect on targeted quantity of *Roseburia* spp., known as an important butyrate producer [68]. Butyrate represents the most preferential energy source of colonocytes [69], resulting in the stimulation of epithelial cell proliferation and mucus secretion [70]. Therefore, the supplementation of *B. subtilis* and *B. licheniformis* may contribute to an improved gut health of pigs.

Assay diets did not significantly impact overall microbiota, but influence was demonstrated for bacterial copy numbers of *Bacteroides*-*Prevotella*-*Porphyromonas*. In the present study, the dietary CP level and the supplementation of *B. subtilis* and *B. licheniformis* showed an interaction, as supplementation of *B. subtilis* and *B. licheniformis* increased *Bacteroides*-*Prevotella*-*Porphyromonas* in the LP diets when compared to HP diets. The *Bacteroides*-*Prevotella*-*Porphyromonas* group includes phylogenetic related species from *Bacteroidetes* phylum that commonly inhabit GIT. Sequencing results confirmed an increased abundance of *Prevotella* in ileal digesta from LP + treatment when compared to the other assay diets. This finding is in agreement with other studies, which showed an enhancing effect of low protein diets on gene copy numbers of *Bacteroides*-*Prevotella*-*Porphyromonas* group in ileal digesta [17], and a significant increase in the abundance of *Prevotella* genus in cecum [71] when compared to samples of treatments with a higher protein level [17, 71]. *Prevotella* dominate the porcine fecal metagenome [72], play an important role in intestinal carbohydrate fermentation [73] and also show proteolytic activity [74]. Sequencing results also revealed members of *Prevotella* as main discriminators of community structure from ileal microbiota of starter and experimental periods. The observed decrease over experimental time is in agreement with a longitudinal study of Kim et al. [75]. Thus, the observations on relative proportion of *Prevotella* represent the general impact of diet and age on porcine intestinal microbiota. Contrary to ileal digesta, where abundance of *Prevotella* was highest in LP +, the fecal proportion of *Prevotella* was higher in HP than LP treatment and slightly increased over experimental time. This variation along sampling sites is in agreement with a previous study, investigating as well ileal digesta and fecal samples from pigs [17], where abundance patterns of *Prevotella* species in the GIT of pigs were different between ileal digesta and fecal samples.

The results of this study demonstrate an overall lower bacterial diversity for ileal digesta compared with fecal samples. Metagenome studies on porcine microbiota collected from different intestine sites revealed different contributions of bacterial species and activities along the GIT [76, 77]. The fecal collection is an easy accessible sampling site with samples showing high similarity to microbiota composition from proximal intestine. However, microbiota composition from fecal samples is not identical representatives to those from ileal digesta. Therefore, collecting samples of different sites of the GIT, where close interactions between the microbiome and the digestive processes occur, will improve understanding of probable functional changes and the effects of dietary treatments such as the addition of probiotics.

Undigested dietary components passing into the large intestine are subjected to fermentation by the intestinal microbiota [17]. As a result, fermentation products such as SCFA are rapidly absorbed across the gut wall, contributing up to 30% of growing pigs’ maintenance requirement for energy [78]. On the other hand, increasing protein fermentation may result in the formation of detrimental fermentation products such as ammonia and amines in the colon [79]. A lower dietary protein level may reduce ammonia production, as observed by Htoo et al. [14] in cecal samples of pigs, while supplementation of diets with *B. subtilis* and *B. licheniformis* showed similar results in slurry samples from pigs due to a lowering effect on the pH [6]. Therefore, LP diets supplemented with *B. subtilis* and *B. licheniformis* might be used to reduce the production of harmful microbial metabolites in the large intestine of pigs.

## Conclusions

Supplementation with *Bacillus* spp. did not affect both AID and SID of CP and AA in growing pigs. The higher SID of some AA in the LP diets when compared to HP diets hints towards the possibility of reducing N excretion through diet manipulation. Regarding microbiota, the assay diets had no significant effect on overall community structure, neither in ileal digesta nor feces. Nevertheless, dietary protein content and *Bacillus* spp. supplementation may enhance various community members in ileal digesta. Within this regard, feeding of the HP diet resulted in a higher abundance of *Lactobacillus* spp. and *Bifidobacterium* spp., whereas LP diet may support bacteria important for carbohydrate degradation such as *Prevotella*. Furthermore, relative proportion of *Prevotella* was altered during pig’s age. The supplementation of *Bacillus* spp. promoted gene copy numbers of *Roseburia* spp., which may be beneficial due to ascribed health promoting properties of this butyrate producer, and this phenomenon may be more effective under stress condition. The LP diet supplemented with *B. subtilis* and *B. licheniformis* may be used as an alternative feeding strategy to support gut health in pigs.

## Abbreviations

AA: Amino acid
ADF: Acid detergent fiber
ADL: Acid detergent lignin
AID: Apparent ileal digestibility
ANOSIM: Analysis of similarity
BW: Body weight
CFU: Colony forming units
CP: Crude protein
DM: Dry matter
GIT: Gastrointestinal tract
HP: High-protein diet
LP: Low-protein diet
ME: Metabolizable energy
N: Nitrogen
NDF: Neutral detergent fiber
OTU: Operativ taxonomic unit
PERMANOVA: Permutational multivariate analysis of variance
qPCR: Quantitative real-time PCR
RELATE: Mantel type test
SCFA: Short chain fatty acids
SID: Standardized ileal digestibility
